# The Influence of Individual Differences on Diverging Behavior at the Weaving Sections of an Urban Expressway

**DOI:** 10.3390/ijerph18010025

**Published:** 2020-12-22

**Authors:** Zhanji Zheng, Qiaojun Xiang, Xin Gu, Yongfeng Ma, Kangkang Zheng

**Affiliations:** 1 Jiangsu Key Laboratory of Urban ITS, Jiangsu Collaborative Innovation Center of Modern Urban Traffic Technologies, School of Transportation, Southeast University, Nanjing 21189, Jiangsu, China; zhengzhanji@126.com (Z.Z.); mayf@seu.edu.cn (Y.M.); zkk_light@163.com (K.Z.); 2 Beijing Key Laboratory of Traffic Engineering, Beijing University of Technology, Beijing 100124, China; guxin@bjut.edu.cn

**Keywords:** weaving section, driving behavior, driver personality, driving simulator, duration time

## Abstract

Urban expressway weaving sections suffer from a high crash risk in urban transportation systems. Studying driving behavior is an important approach to solve safety and efficiency issues at expressway weaving sections. This study aimed to investigate the influence of drivers’ individual differences on diverging behavior at expressway weaving sections. First, a k-means cluster analysis of 650 questionnaires was performed, to classify drivers into three categories: aggressive, conservative and normal. Then, the driving behavior of 45 drivers from the three categories was recorded in a driving simulator and analyzed by an analysis of variance. The results show that different types of drivers have different driving behaviors at weaving sections. Aggressive drivers have a higher mean speed and mean longitudinal deceleration, followed by normal and conservative drivers. Significant differences in the range of lane-change positions were found between 100, 150 and 200 m of weaving length for the same type of drivers, and the duration of weaving for aggressive drivers was significantly smaller than for normal and conservative drivers. A significant correlation was found between lane-change position and weaving duration. These results can help traffic engineers to propose effective control strategies for different types of drivers, to improve the safety of weaving sections.

## 1. Introduction

Numerous expressways have been constructed in metropolises, to reduce travel time and improve traffic capacity. Urban expressways play an important role in daily urban transportation throughout the road network, in the context of urban development. However, evidence has shown that weaving sections, as an important part of the expressway, are more likely to be crash-prone areas, as compared to the ordinary road (Golob et al., 2004; Hidas, 2005; and Mao et al., 2019) [1,2,3]. One possible reason for this is that the driver needs to conduct mandatory lane-changing behaviors in a limited distance, to get into his/her target lanes in the weaving section when a merging area is closely followed by a diverging area (Wang et al., 2016; Ali et al., 2019; Luo et al.,2019; and Hao et al., 2020) [4,5,6,7].

In addition, the human factor is the most important component in the transportation system, which is composed of the driver–vehicle–road–environment interaction. Lane-changing behavior is a dynamic process that requires drivers to acquire and process information and operate the vehicle in real time. Therefore, the performance of lane-changing behavior is affected by driver characteristics, such as age, gender, attitudes and aggressiveness levels, which means that this behavior differs greatly among driver types (Rong et al., 2011; Hill et al., 2015; and Lyu et al., 2018) [8,9,10]. The main objective of this paper is to analyze lane-changing behaviors at weaving sections and to investigate the relationships between different types of drivers and their lane-changing characteristics.

## 2. Literature Review

### 2.1. Risk Factors of Traffic Accidents at Weaving Sections

Numerous studies have been conducted to identify the important risk factors for traffic accidents at weaving sections (Cirillo, 1970; Fazio et al., 1993; Glad, 2001; and Golob et al., 2004) [1,11,12,13]. The geometric design of weaving sections is an important factor that impacts safety. Pulugurtha (2010) [14] studied the relationship between crashes and weaving-section characteristics and traffic variables. It was found that weaving characteristics and traffic variables had a significant influence on crashes. An increase in the length of weaving sections was found to cause a decrease in crashes; besides this, an increasing exit volume was the main factor causing an increase of rear-end crashes, as well as crashes due to following too closely and inattentive driving accidents. In addition, weather conditions, traffic and driver characteristics are also considered to be associated with traffic-crash risks. Wang et al. (2015) [15] analyzed the crashes at expressway weaving segments by developing a multilevel Bayesian logistic regression model. It was found that speed differences played an important role in the crash risk at weaving sections. A wet-pavement surface condition and increased difference of speed were found to significantly increase the crash ratio. Penmetsa and Pulugurtha (2018) [16] developed a multinomial logit model to investigate the effect of road features on crash injury severity. They found that driver age and gender significantly affect crash-injury severity and crash frequency at weaving sections.

### 2.2. Characteristics of Driving Behavior at Weaving Sections

In previous research, several studies have been conducted to investigate the characteristics of driving behavior at weaving sections. Skabardonis and Kim (2010) [17] found that mandatory lane-changing behaviors at weaving sections were influenced by traffic conditions in the auxiliary lane, and most lane-changing behaviors occurred in the area between the curbside lane and the auxiliary lane. Du et al. (2013) [18] used video data to investigate the lane-changing behavior on freeways and found that more lane-changing behavior occurred and shorter time gaps were accepted when drivers in the limited-access high-occupancy vehicle (HOV) lanes exit HOV lanes. Sarvi (2013) [19] developed a theoretical framework for modeling a weaving driver’s acceleration–deceleration behavior by using the psychophysical concept of stimuli–response. It was found that the weaving vehicles’ acceleration behaviors were significantly different from non-weaving vehicle drivers’ behaviors and were significantly affected by the surrounding vehicles at weaving sections. Kusuma et al. (2015) [20] analyzed the lane-changing characteristics at weaving sections by using a loop detector and video-recording data and found that multiple lane-changing drivers were more aggressive compared to the single lane-changing drivers at the moment of their first lane change due to the multiple lane-changing drivers accepting smaller headways. Blasiis et al. (2017) [21] analyzed drivers’ weaving maneuvers through deceleration and risk area indicators by using a driving simulator. The results showed that drivers’ deceleration behaviors were significantly affected by the traffic flow in the main lane. Van Beinum et al. (2018) [22] found that most lane-changing behaviors of merging and diverging vehicles make use only of the first 25% of the weaving section, and only a few lane-changing behaviors occurred in the last part of weaving section. Yuan et al. (2019) [23] investigated the safety effects of drivers’ lane-changing behaviors with a driving simulator. They found that lane-changing that occurred in the last lane had the shortest duration time on average of all lane changes, and the duration of the average lane change at the entrance of the weaving section was slightly longer than that on the exit of the weaving section for the driving simulator.

### 2.3. Classification of Driver Type

The objective experimental data from actual driving experiments or driving simulators were used to classify driver types in previous research. Aljaafreh et al. (2012) [24] proposed a driving performance inference system that could classify a driver as below normal, normal, aggressive and very aggressive by using their signature of acceleration in two dimensions, as well as their speed. Vaitkus et al. (2014) [25] proposed a method for identifying driving style as aggressive or normal by using three-axis accelerometer data. Rong (2011) [8] investigated the relationship between driver behavior characteristics and traffic flow. A total of 15 indicators were chosen to classify drivers as aggressive, conservative and moderate by using cluster analysis. Sun et al. (2011 and 2014) [26,27] analyzed driver’s perceptions and attitudes regarding lane-changing behaviors by using a focus group study. Participating drivers were classified into four groups, according to driver background data, and behavior data of lane-changing scenarios on urban arterials were gathered by using cluster analysis. Hill (2015) [9] analyzed the relationship between different driver types and lane-changing behaviors. Participating drivers were classified into four groups, according to the data of each participant concerning their driving behavior, the desired speed of the driver and the number of lane changes, by a survey.

In summary, studies centered on different types of drivers’ diverging behaviors at weaving sections are still rare. Lane-changing behavior is not only affected by the external environment (e.g., traffic flow, construction and vehicle performance), but also by the driver’s personal attributes (e.g., habits and aggressiveness level). However, traditional vehicular data only provide basic vehicular information, with no information regarding driver characteristics, which is insufficient to represent the driver’s thinking processes during the maneuvers. Moreover, considering the traffic condition and different driving behaviors of Chinese drivers (Zhang, 2006) [28], it is important to classify drivers by combining driving behavior, vehicle status and driving environment in a customized questionnaire for the specific scene of urban-expressway diverging sections. In this study, we aimed to analyze lane-changing behaviors during vehicle diversion at weaving sections and to investigate the relationships between different types of drivers and their lane-changing characteristics. To this end, drivers were classified based on their background information (age, gender and personality factor) and responses (driving behavior at weaving sections, driving attitude and driving skill) during scenario analysis. Then, a driving simulator experiment was introduced, to obtain driving behavior factors (e.g., acceleration, speed and lane-change position). Factors with significant effects on drivers’ lane-changing behaviors and safety during vehicle diversion are identified in this paper. Finally, the relationships between different types of drivers and their lane-changing characteristics were analyzed based on the results.

## 3. Methodology

### 3.1. Survey and Respondents

In total, 650 questionnaires were sent out at the Department of Vehicle Inspection and Automobile Sales Service Shop, of which 607 were retrieved, representing a recovery rate of 93.4%. All the selected participants owned a car registration document and had over 1 year of driving experience. After excluding questionnaires with missing data or conflicted answers, 583 valid questionnaires were finally obtained, and the valid rate was 89.7%. The details of the participants’ demographics are shown in Table 1.

The questionnaire included 60 questions divided into five main categories: (1) driver general information, as shown in Table 1; (2) driving behavior at weaving sections that was based on the questionnaire and represented lane-changing behavior and scenario interaction (Sun, 2011) [26], and special driving behavior in China (Bai, 2010) [29], which focused on speeding, lane-changing, emergency braking and so on, measured on a five-point Likert scale, ranging from 1 = “never” to 5 = “always” (question No. A1~A18); (3) driving attitude, which focused on individual differences in driving attitudes that were associated with dangerous driving behaviors and traffic accidents (Ulleberg, 2003) [30] and was measured on a five-point Likert scale, ranging from 1 = “strongly disagree” to 5 = “strongly agree” (question No. B1~B11); (4) driving skill, which was based on the revised Driving Skill Inventory (DSI) regarding the relationship between driving skill and accidents (Xu, 2018) [31], and where the situation of scene changing and maintaining vehicle distance was the main focus, which was measured on a five-point Likert scale, ranging from 1 = “strongly agree” to 5 = “strongly disagree” (question No. C1~C12); and (5) personality factor, which concerned the relationship between driving behaviors and personality factors (Liu, 2019) [32] and was measured on a five-point Likert scale, ranging from 1 = “not at all” to 5 = “absolutely” (question No. D1~D10). The estimated time for a participant to complete the questionnaire was about 15 min. The Cronbach’s α was used to analyze the reliability of the questionnaire, and the validity was assessed by the Kaiser–Meyer–Olkin (KMO) test and the results of Bartlett’s spherical test, as shown in Table 2.

### 3.2. Factor Analysis and Cluster Analysis of Driver Classification

The factor analysis was used to clarify the factor structure of each category in the questionnaire. Since the variables in the questionnaire were somewhat related to one another, the factor analysis was used to transform the correlated variables in the questionnaire into a small number of uncorrelated variables. Then, the eigenvalues greater than 1 were selected. The factor structures for each category are shown in Table 3. All analyses were performed with SPSS 15.0, a data mining, statistical and predictive analysis software package. A total of 13 factors were developed from the factor analysis, which could better reflect the driver’s characteristics in terms of driving behavior, driving attitude, driving skills and personality characteristics in weaving sections.

The k-means algorithm was used to cluster participants in this study. The k-means algorithm selected at random some points as the initial clustering center and then minimized the distance from each sample to the clustering center; finally, each data point was assigned to the closest cluster. The aim of the k-means algorithm is to minimize the variance of all scale categories, as shown in Equation (1).

(1) $$J\left(C\right)=\sum_{i=1}^{k}\sum_{x_{i}\in C_{k}}{\left\|x_{i}-\mu_{k}\right\|}^{2}$$

where *μ_k_* is the *k*th clustering center in the *i*th calculation, *x_i_* is the *i*th sample in the class of the *k*th clustering center and *C_k_* is the sample set in the class of the *k*th clustering center.

**Figure 1:**
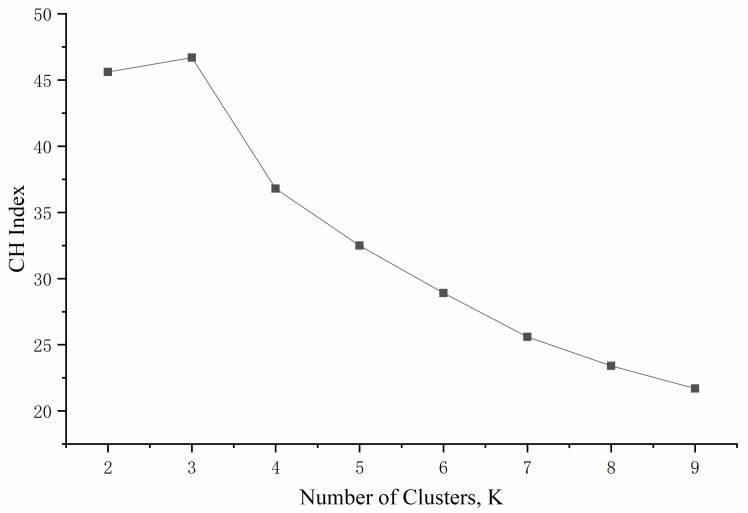
The number of clusters, using the Calinski–Harabasz (CH) index.

The 13 factors developed above were used to classify the drivers by the Calinski–Harabasz (CH) index. The optimal number of clusters occurs when the CH index is 3, which is true for the largest cluster, as shown in Figure 1 (Gao, 2019) [35]. According to the rule of five-point Likert scales, the questionnaire scores of the three types of drivers were counted, and the result is shown in Table 4. Generally speaking, a higher score indicated that it is easier to show an aggressive driving tendency through the question scoring rules. The questionnaire score was consistent with the trend of the number of accidents and accidents with injured persons.

### 3.3. Experimental Scene Development

**Figure 2:**
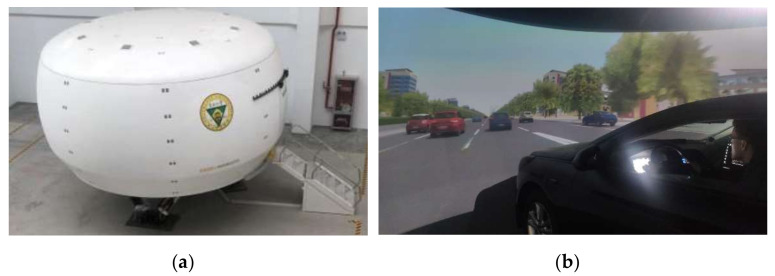
(**a**) Driving simulator; (**b**) screenshot in driving simulator.

A driving simulation enables researchers to study driving behavior in a safe and highly controllable (virtual) environment. The Southeast University driving simulator was used to perform the driving simulation, as shown in Figure 2. The simulator consists of two main components: the driving simulation cabin is mounted on a six-degree-of freedom motion system, and the visual simulation system provides a view of a 360° driving environment with a resolution of 1400 × 1050 pixels and a 60 Hz refresh rate. LCD (Liquid Crystal Display) monitors provided rear views at the central and side mirror positions.

**Figure 3:**
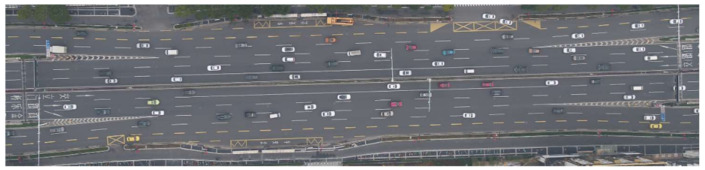
Aerial view of the urban expressway at a weaving section.

**Figure 4:**
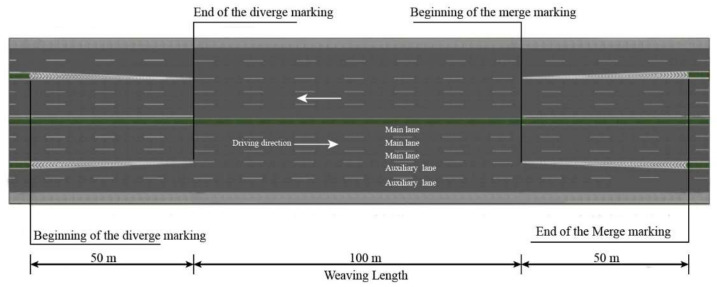
Schematic diagram of the weaving section.

The simulated road was based on the weaving section of the urban expressway in Longpan Road, Nanjing—a typical weaving section in Jiangsu Province, China—as shown in Figure 3. The surveys were conducted in October 2019, before the morning and evening peak periods (7:00–7:30 and 16:30–17:00) of the weaving section. The traffic condition was recorded by an unmanned aerial vehicle (UAV) and used for the simulation scene: The average travel speed was 45.6 km/h, the volume/capacity ratio was 0.55:1 and the density was 20 to 30 passenger-car units per kilometer per lane, of which 87.4% were passenger cars, 10.4% were midsized vehicles and 2.2% were large vehicles. The urban expressway was characterized by three driving lanes in each direction on the main lane and two driving lanes on the auxiliary lane, with a lane width of 3.75 m. The shoulder width accounted for 3 m, and the center median separating the driving directions was 2 m. The width of separation between the main lane and auxiliary lane was 2 m (see Figure 4).

In total, an 8 km long simulation drive was created. Four weaving sections were implemented at varying locations (1, 3.5, 5 and 7 km) along the drive. The distances between weaving sections were long enough to ensure that there was no overlap in the influence areas of weaving sections.

### 3.4. Participants and Experimental Procedure

Forty-five drivers were recruited for this study, including 32 males and 13 females, ranging in age from 21 to 64 years (mean = 37.5; SD = 11.6). To resemble the driver type composition in this study, the participants were selected from those who participated in the questionnaire survey, by a random sampling approach, randomly selecting 15 drivers from each of the three types of drivers.

Before the formal test, the participants were required to perform a practice drive for 5~10 min, with the help of operators. All participants had to confirm that they felt sufficiently familiar with the simulator before starting the formal test. Then, the formal test followed, where each participant drove all four weaving sections in the same order. In this study, we were mainly interested in the diverging behavior of participants in the main lane when moving through a weaving section on the urban expressway. Therefore, participants were required to complete lane-change operations from the main lane to auxiliary lane in any of the four weaving sections. It took about 15 min to complete the formal test. Afterward, additional information was collected via a post-simulation survey, to ask drivers about their feelings regarding the experiment. The results indicated that almost all of the participants said the realism of the simulator system and the road scenarios were good, and none of the drivers felt sick during the experiment. These tests were conducted with the consent of all participants. In addition to ensuring the safety of the drivers, we also guaranteed that participants’ information was protected and kept confidential.

## 4. Data Analysis and Result

A set of driving behavior parameters was analyzed: mean speed (in km/h), mean longitudinal acceleration (s/m^2^) and the standard deviation of lateral acceleration (m/s^2^) (SD of lateral acceleration) (Ariën, 2013; Reinolsmann, 2019) [36,37]. Besides this, the lane-change position and lane-changing duration time were measured and analyzed (Toledo, 2007) [38]. Sixty-one measurement points were analyzed in 5 m intervals over a travel distance of 50 m before the beginning of the diverge marking to 50 m after the end of the merge marking. The beginning of the diverge marking, end of the diverge marking, beginning of the merge marking and end of the merge marking were indicated by a gray vertical dotted line at points of 50, 100, 200 and 250 m, respectively. The parameters were analyzed by one-way analysis of variance (ANOVA). For all analyses, the significance level was set at 0.05.

### 4.1. Mean Speed

**Figure 5:**
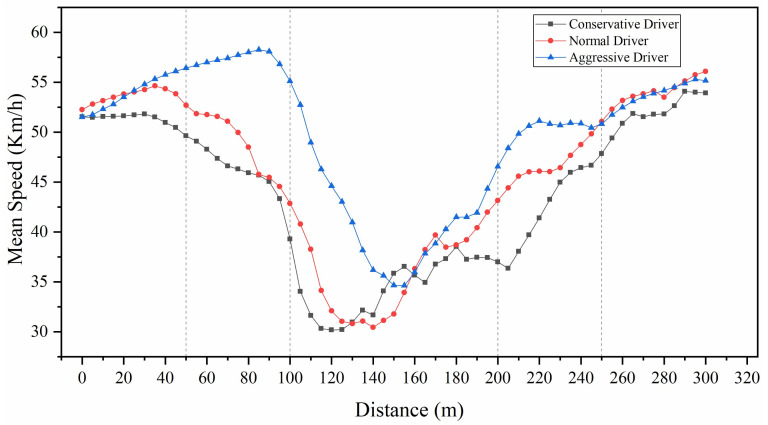
Mean speed for three type of drivers.

Figure 5 shows the mean speed for each type of driver in 5 m intervals over a travel distance of 50 m before the beginning of the diverge marking to 50 m after the end of the merge marking. The results in Figure 5 reveal the following:

(1) For a mean speed in the range of 0 to 300 m, the aggressive drivers had the highest mean speed (mean = 49.75 km/h, SD = 5.04 km/h), followed by normal drivers (mean = 46.04 km/h, SD = 3.32 km/h) and conservative drivers (mean = 43.60 km/h, SD = 3.85 km/h) (*F*(2,42) = 11.629, *P* < 0.01). The aggressive drivers had the highest speed at the point at 85 m. The conservative drivers had the lowest speed at the point at 120 m.

(2) An analysis of variance was performed regarding the mean speed at points of 0~300 m. The results showed that, for the points at 0~35 m, no significant differences in mean speed were shown for the three types of drivers. The drivers started to increase speeds in this area. The conservative drivers increased speeds (up to 51.81 km/h) until the point at 30 m, and the normal drivers increased their speeds (up to 54.64 km/h) until the point at 35 m. For the points at 40~130 m, there were significant differences in the mean speeds of different types of drivers. The operations began to change significantly between different drivers. The conservative drivers and normal drivers began to decrease speeds in order to prepare for the diversion behavior of vehicles in the weaving section, while the vehicle gradually completed the deceleration process. The speed of conservative drivers reached its lowest level at the point at 120 m. In contrast, the aggressive drivers continued to increase their speeds (up to 58.26 km/h) until the point at 85 m and then began to decrease speeds. For the points at 135~180 m, there was no significant difference in mean speeds of the different types of drivers. The speed of normal drivers and aggressive drivers reached the lowest levels at the points of 140 and 155 m, respectively. All types of drivers completed the lane-changing process in this area, and the speeds began to increase gradually. For the points at 185~220 m, there were significant differences in the mean speeds of different types of drivers. All types of drivers increased their speeds; however, the speed difference between different types of drivers gradually decreased. For the points at 225~300 m, there were no significant differences in the mean speeds of different types of drivers, as the vehicle was about to leave the weaving section and the speed of different types of drivers tended to be stable.

### 4.2. Mean Longitudinal Acceleration

**Figure 6:**
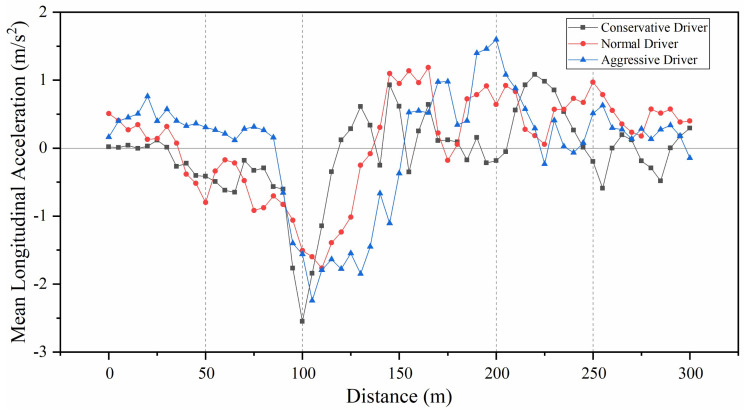
Mean longitudinal acceleration for three types of driver.

Figure 6 shows the mean longitudinal acceleration for each type of driver in 5 m intervals over a travel distance of 50 m before the beginning of the diverge marking to 50 m after the end of the merge marking. The results in Figure 6 are as follows.

An analysis of variance was performed for mean longitudinal acceleration in the sections of 0~50 m, 50~100 m, 100~200 m, 200~250 m and 250~300 m. The results showed that, in the range of 0~50 m, the mean longitudinal acceleration showed significant differences for three types of drivers (*F*(2,30) = 10.01, *P* < 0.01). The deceleration behaviors of normal drivers and conservative drivers started to occur in this area; however, the aggressive drivers showed acceleration behavior. In the range of 50~100 m, there were significant differences in the mean longitudinal acceleration of different types of drivers (*F*(2,27) = 3.45, *P* = 0.04 < 0.05). The deceleration behavior of conservative drivers started to appear at the point of 90 m, while normal drivers and aggressive drivers were already in a deceleration state. In the range of 100~200 m, while generally in a state of decelerating first and then accelerating, the acceleration of aggressive drivers was first greater than 0 (at a point of 120 m), followed by normal drivers (at a point of 140 m) and conservative drivers (at a point of 155 m); however, there were no significant differences in the mean longitudinal acceleration of different types of drivers in this area (*F*(2,57) = 0.69, *P* = 0.51 > 0.05). In the range of 200~250 m, different types of drivers were in a state of accelerating; however, there were no significant differences in the mean longitudinal acceleration rates of different types of drivers in this area (*F*(2,57) = 3.01, *P* = 0.06 > 0.05). In the range of 250~300 m, the mean longitudinal acceleration showed significant differences for the three types of drivers (*F*(2,27) = 13.32, *P* < 0.01).

### 4.3. SD of Lateral Acceleration

**Figure 7:**
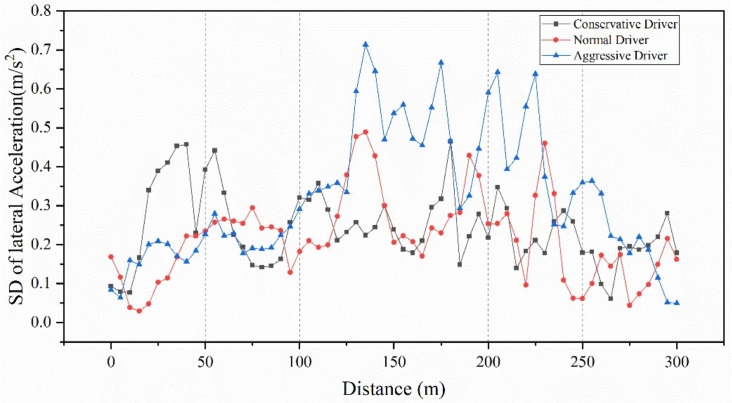
Standard deviation (SD) of lateral acceleration for three types of driver.

The SD of lateral acceleration was calculated based on 5 m segments and plotted to analyze the homogeneity of lateral acceleration among drivers who conducted lane changes at weaving sections, as shown in Figure 7, and showed the following results.

An analysis of variance was performed regarding the SD of lateral acceleration in the sections of 0~50 m, 50~100 m, 100~200 m, 200~250 m and 250~300 m. The results showed that, in the range of 0~50 m, the SD of lateral acceleration showed significant differences for the three types of drivers (*F*(2,30) = 6.22, *P* < 0.01). The conservative drivers had the largest SD of lateral acceleration compared to the other types of drivers; this could be due to the conservative drivers starting to adjust lanes earlier than normal and aggressive drivers to prepare for lane changes in the weaving section. In the range of 50~100 m, there were no significant differences in the SD of lateral acceleration of different types of drivers (*F*(2,27) = 0.11, *P* = 0.89 > 0.05). In the range of 100 m~200 m, substantially higher values for SD of lateral acceleration were measured for the aggressive drivers compared to normal and conservative drivers (*F*(2,57) = 25.46, *P* < 0.01). This indicates that there were high variations in aggressive drivers’ lateral acceleration at 130~175 m. In the range of 200~250 m, the SD of lateral acceleration of aggressive drivers was significantly higher than normal and conservative drivers (*F*(2,27) = 8.82, *P* < 0.01). This can be explained by aggressive drivers continuously trying to find gaps to adjust their lane to increase their speed as quickly as possible. In the range of 250~300 m, there were no significant differences in the SD of lateral acceleration of different types of drivers (*F*(2,27) = 1.68, *P* = 0.20 > 0.05). This could be due to the vehicles tending to be stable after leaving the weaving section.

### 4.4. Lane-Change Position and Duration Time

**Figure 8:**
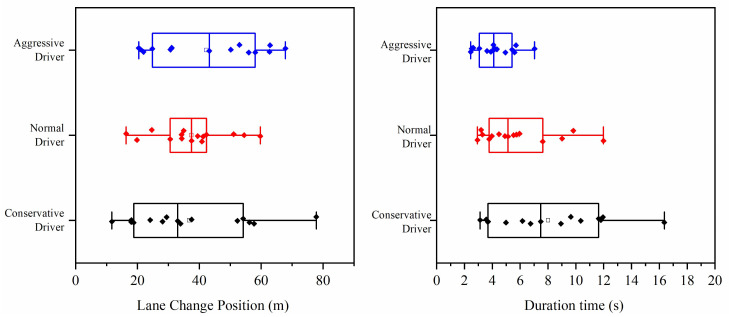
Lane-change position and duration time for three types of driver.

The lane-change position was calculated by the distance between the point where the vehicle changed lanes from the main lane to auxiliary lane and the end of the diverge marking. The lane-change position and duration time of each participant for the three types of drivers are plotted in Figure 8. The results in Figure 8 are as follows.

The conservative drivers had the largest range of lane change position (mean = 36.72, SD = 18.28), followed by the normal drivers (mean = 37.43, SD = 12.01) and aggressive drivers (mean = 42.31, SD = 16.63). It seems that the aggressive drivers had a more backward lane-change position than normal drivers and conservative drivers; however, the analysis of variance showed that there were no significant differences in the lane-change position of different types of drivers (*F*(2,42) = 0.58, *P* = 0.56 > 0.05).

The conservative drivers had the largest range of durations of lane changes (mean = 8.01, SD = 3.81), followed by the normal drivers (mean = 5.82, SD = 2.58) and aggressive drivers (mean = 4.30, SD = 1.21). The analysis of variance showed that there were significant differences in the duration of lane changes for different types of drivers (*F*(2,42) = 6.44, *P* < 0.01). This indicates that aggressive drivers take less time to change lane than normal drivers and conservative drivers.

**Figure 9:**
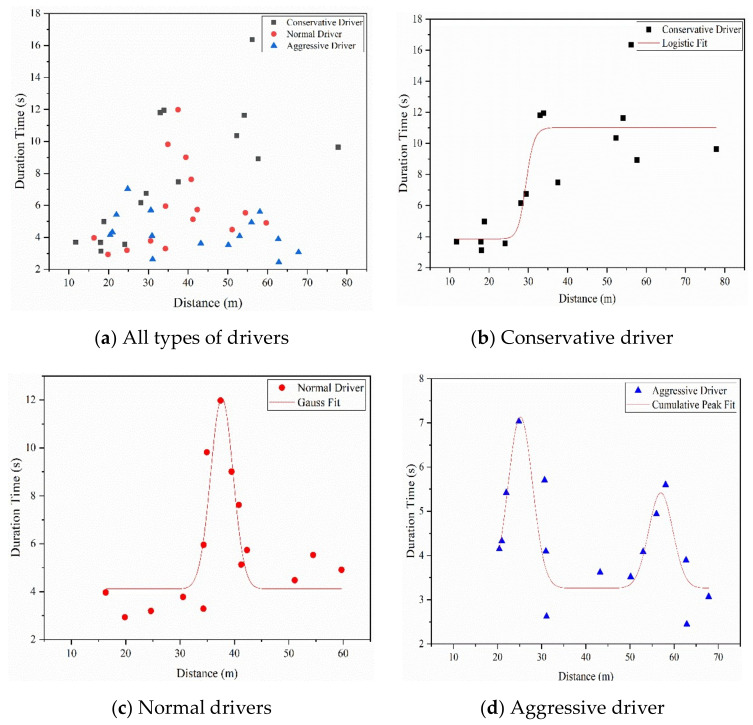
The relationship between lane-change position and duration.

Furthermore, the figure of lane-change position–duration time was plotted to gain deeper insights into the relationship between lane change position and duration. The logistic curve fitting method was used for the conservative drivers’ data, and the Gaussian curve fitting method was used for the normal drivers’ and aggressive drivers’ data, as shown in Figure 9b–d, respectively. The logistic fitting equation was expressed as follows:

(2) $$y=\left(A_{1}-A_{2}\right)/\left(1+{\left(x/x_{0}\right)}^{p}\right)+A_{2},$$

The Gaussian fitting equation was expressed as follows:

(3) $$y=y_{0}+\frac{A}{w\sqrt{\pi /2}}\exp\left(-2{\left(x-x_{c}\right)}^{2}/w^{2}\right),$$

The coefficients for the fitting equation are listed in Table 5.

In Equation (2), *x*_0_ represents the turning point of the lane change duration from fast to slow (Zheng, 2017) [39]. In Equation (3), *x*_c_ represents the expected value of the distance. The maximum duration time occurs at *x* = *x*_c_. From Table 5 and Figure 9, we find the following:

(1) For conservative drivers, the lane-change position was uniformly distributed within the range of 10~80 m. The duration of changing lane increases as the lane-change position distance increases. Based on *x*_0_, the duration time will change from low to high.

(2) For normal drivers, the lane-change position was mainly concentrated within the range of 30~45 m. The duration of changing lane obeys a Gaussian distribution and reaches a peak at *x*_c_ = 37.69 m and then decreases.

(3) For aggressive drivers, the lane-change position was mainly concentrated at the two ends, in the range of 20~30 m and 50~70 m, respectively. The duration of changing lanes had two peaks. Based on *x*_c_, the duration reaches the first peak at *x*_c_ = 25.67 m and the second peak at *x*_c_ = 57.14 m.

## 5. Discussion

Lane-changing is an important aspect of driver behavior that has a significant impact on the weaving section operation. One effective way to improve the safety and efficiency of weaving sections is to better understand and integrate driver behavior so that the weaving section structure can better adapt to drivers’ behaviors. To achieve this aim, three kinds of weaving length (100, 150 and 200 m) were designed. The effects of the weaving-length factor on different types of drivers’ lane-changing behaviors are fully discussed in the following subsections.

### 5.1. Mean Speed and Mean Maximum Longitudinal Deceleration

**Figure 10:**
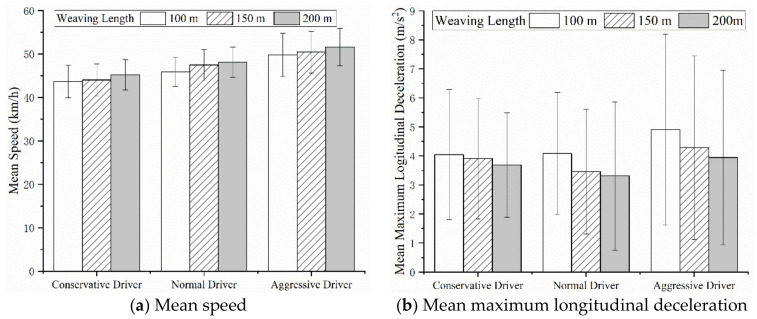
Mean speed and mean maximum longitudinal deceleration for different weaving lengths.

As shown in Figure 10, aggressive drivers showed a higher mean speed and mean longitudinal deceleration, followed by normal and conservative drivers, which is similar to the results of previous studies (Fuller, 2005; Sagberg, 2015) [40,41]. With respect to mean speed, all types of drivers’ mean speeds increased as the weaving length increased; however, no significant difference were found in mean speeds between 100, 150 and 200 m of weaving length for the same type of drivers (*F*_C_(2,42) = 0.713, *P* = 0.496 > 0.05; F_N_(2,42) = 1.713, *P* = 0.193 > 0.05; *F*_A_(2,42) = 0.574, *P* = 0.568 > 0.05). Meanwhile, the one-way ANOVA showed a significant difference in mean speed between different types of drivers under the same weaving length (*F*_100_(2,42) = 8.425, *P* < 0.01; *F*_150_(2,42) = 9.163, *P* < 0.01; *F*_200_(2,42) = 0.574, *P* < 0.01). With respect to the mean maximum longitudinal deceleration, all types of drivers’ mean maximum longitudinal deceleration decreased as the weaving length increased; however, no significant differences were found in mean maximum longitudinal deceleration between 100, 150 and 200 m of weaving length for the same type of drivers (*F*_C_(2,42) = 0.528, *P* = 0.593 > 0.05; *F*_N_(2,42) = 0.410, *P* = 0.666 > 0.05; *F*_A_(2,42) = 0.244, *P* = 0.784 > 0.05) or between different types of driver under the same weaving length (*F*_100_(2,42) = 0.117, *P* = 0.890 > 0.05; *F*_150_(2,42) = 0.493, *P* = 0.614 > 0.05; *F*_200_(2,42) = 0.362, *P* = 0.698 > 0.05). It seems that a longer weaving length might increase the risk of a crash, due to higher driving speed and deceleration (Reinolsmann, 2019; Ma,2020) [37,42]. This result is in agreement with a previous study by Yuan (Yuan, 2019) [23]. It could be that a long weaving length might cause drivers to behave in an uncertain manner (De Blasiis, 2017) [21].

### 5.2. Lane Change Position and Duration

**Figure 11:**
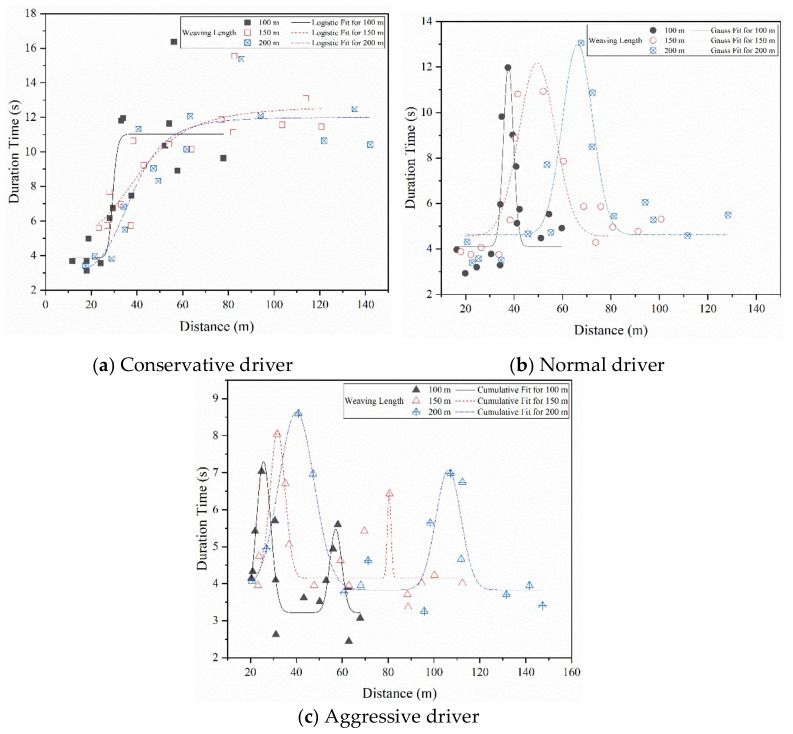
Lane-change position and duration at different weaving lengths.

With respect to lane change position, it is obvious that all types of drivers’ ranges of lane-change positions increased as the weaving length increased; statistically significant differences in the ranges of lane-change positions were observed between 100, 150 and 200 m of weaving length for the same type of drivers, by the ANOVA method (*F*_C_(2,42) = 3.465, *P* = 0.04 < 0.05; *F*_N_(2,42) = 4.750, *P* = 0.01 < 0.05; *F*_A_(2,42) = 7.346, *P* < 0.01). As shown in Figure 11, the lane-change position of conservative drivers was mainly distributed within around the first 80% of the weaving length. The lane-change position of normal drivers was mainly concentrated within around the first 60% of the weaving length, while the lane-change position of aggressive drivers was mainly concentrated around the first 70% of the weaving length. This result indicated that lane-changing to the auxiliary lane is performed by diverging vehicles sufficiently far before the beginning of the merge marking, which is in line with previous research (Zhang, 2018) [43].

With respect to the duration of lane changes, it is obvious that the conservative drivers had the largest range of duration time for the three kinds of weaving length, followed by normal drivers and aggressive drivers. Moreover, it was found that the aggressive drivers had a smaller duration of lane-changing compared to normal and conservative drivers, which is similar to the findings of previous studies (Hill, 2015; Shi, 2017) [9,44]. As shown in Figure 11, the lane-changing duration of conservative drivers in the posterior segment of the lane change position was higher than in the anterior segment. The duration of normal drivers in the intermediate section was higher than at the two ends; this situation stands in contrast to the aggressive drivers’ duration, which was higher at the two ends. The ANOVA showed a significant difference in duration between different types of drivers under the same weaving length (*F*_100_(2,42) = 6.442, *P* < 0.01; *F*_150_(2,42) = 18.30, *P* < 0.01; *F*_200_(2,42) = 8.239, *P* < 0.01). The findings reveal the mechanism of lane-changing behavior of different types of drivers at weaving sections. These can help improve driveway management at weaving sections and provide a reference for the layout of guide signs.

## 6. Conclusions

Driving-simulation experiments were carried out in this study, to investigate lane-changing behaviors at weaving sections, and were used to examine the relationships between different types of drivers and their lane-changing characteristics. Firstly, a cluster analysis was performed to classify drivers into three categories: aggressive drivers, conservative drivers and normal drivers. Secondly, the driving behavior parameters for the three types of drivers were collected by using a driving simulator. Then, the relationships between different types of drivers and their lane-changing characteristics were analyzed. Finally, the effects of the weaving length factor on different types of drivers’ lane-changing behaviors were investigated. The following conclusions could be drawn.

Different types of drivers have different driving behaviors at weaving sections. For a weaving length of 100 m, aggressive drivers have the largest mean speed, followed by normal drivers and conservative drivers. There are no significant differences in the mean longitudinal acceleration of different types of drivers; however, the SD of lateral acceleration of aggressive drivers is significantly higher than that of normal and conservative drivers. There are no significant differences in the lane-change position of different types of drivers; however, the lane-changing duration of aggressive drivers is significantly smaller than normal and conservative drivers.

With respect to the effect of weaving length, as the weaving length increases, all types of drivers’ mean speeds and ranges of lane-change positions increase, while the mean maximum longitudinal deceleration decreases; however, no significant differences were found in terms of mean speed and mean maximum longitudinal deceleration between the three kinds of weaving length for the same type of drivers. The lane-change positions of all types of drivers were distributed within an area not exceeding the first 80% of the weaving length.

A significant correlation was found between lane-change position and duration. Via logistic function fitting, the duration of conservative drivers in the posterior half of the lane-change position was found to be higher than in the anterior half. Via Gaussian function fitting, the duration of normal drivers in the intermediate section of the lane-change position is higher than at the two ends; however, the duration of aggressive drivers was found to be higher at the two ends via a multimodal Gaussian fitting.

This study also has some methodological limitations. Firstly, the respondents were selected at the local Department of Vehicle Inspection and Automobile Sales Service Shop, which may lead to the possibility of sampling bias. Meanwhile, the respondents had different backgrounds, which may have caused the respondents to have different understandings of the same questions in the questionnaire. This was affected by the implementation capacity of investigators. Secondly, the present study simulated the average traffic density at weaving sections on the Nanjing urban expressway, and further research is needed to test drivers’ interactions with different numbers of lanes and different environments in different traffic conditions.

## Figures and Tables

**Table 1:** Participant demographics (*n* = 583).

Driver Information		N	Percent (%)
Gender	Male	457	78.3
	Female	126	21.6
Age	18~30	219	37.6
	31~40	205	35.2
	41~50	107	18.4
	>50	52	8.9
Working as a driver	Yes	154	26.4
	No	429	73.6
Whether a vehicle commuter or not	Yes	340	58.3
	No	243	41.7
Annual mileage (km)	≤10,000	79	13.6
	10,001~50,000	92	15.8
	50,001~100,000	107	18.4
	100,001~300,000	161	27.6
	>300,000	144	24.7
Driving years	≤3	121	20.8
	4~5	106	18.2
	6~10	186	31.9
	11~15	88	15.1
	>15	82	14.1
Vehicle type	Passenger Car	402	69.00
	SUV	99	17.00
	MPV	10	1.70
	Other	72	12.30
Ran a red light within last 3 years	0	254	43.6
	1~2	234	40.1
	3~4	71	12.2
	≥5	24	4.2
Whether accidents happened within the last 3 years	Yes	169	29.0
	No	414	71.0
Whether anyone was injured in an accident within the last 3 years	Yes	26	4.5
	No	557	95.5

Note: The vehicle type was classified as passenger car, sport utility vehicle (SUV), multi-purpose vehicle (MPV) and cross-passenger car (Zheng, 2019) [33], where the cross-passenger car was represented by “others”.

**Table 2:** The reliability and validity test of questionnaire.

Factor	Cronbach’s α	KMO	Bartlett’s Spherical Test, Sig Level = 0.05
Driving behavior at weaving sections	0.866	0.816	0.000
Driving attitude	0.859	0.793	0.000
Driving Skill	0.942	0.842	0.000
Personality	0.837	0.708	0.000

Note: Cronbach’s α > 0.70 for factors was acceptable (Sun, 2019) [34]. A Kaiser–Meyer–Olkin (KMO) value >0.7 shows a strong correlation between the observed variables (Sun, 2019) [34].

**Table 3:** Factor structure.

Category	Factor	Question No. Group
1	Emotional factor	A11, A10, A16, A15, A18
	Speeding factor	A8, A7, A3, A17
	Negligence factor	A4, A5, A1, A2
	Wrong driving factor	A4, A14, A13
	Risk factor	A12, A9, A6
2	Ignore speed factor	B5, B7, B1, B6
	Ignore rule factor	B11, B2, B3, B4
	Entertainment factor	B8, B9, B10
3	Normal operating attitude factor	C7, C8, C6, C9, C11, C3, C2, C12, C10, C1
	Special operating attitude factor	C4, C5
4	Irritability factor	D7, D6, D8, D5
	Environmental adaptability factor	D4, D3, D2, D1
	Pursuit stimulus factor	D10, D9

Note: The question-number group is sorted by factor loadings from largest to smallest.

**Table 4:** The result of the driver classification.

Parameters	Type A	Type B	Type C
N	160	186	237
Percent (%)	27.4	31.9	40.7
Mean score in questionnaire	145.6	118.9	131.1
Median score in questionnaire	145	121	129
Define the driving style	Aggressive	Conservative	Normal
No. of accidents	73	39	57
No. of accidents with injured person	18	3	5

**Table 5:** The coefficients of curve fitting.

**Logistic Fitting**		***A*** **_1_**	***A*** **_2_**	***x*** **_0_**	***p***	**Adj. R^2^**
Conservative drivers		3.862	11.036	29.425	28.633	0.6876
**Gauss Fitting**		***A***	***w***	***x*** **_c_**	***y*** **_0_**	**Adj. R^2^**
Normal driver		40.988	4.103	37.690	4.112	0.714
Aggressive drivers	Peak1	31.506	6.162	25.671	3.217	0.539
	Peak2	14.609	5.167	57.148	3.217	

## Data Availability

The data presented in this study are available on request from the corresponding author. The data are not publicly available due to privacy.
